# The Role of B-cells and IgM Antibodies in Parasitemia, Anemia, and VSG Switching in *Trypanosoma brucei*–Infected Mice

**DOI:** 10.1371/journal.ppat.1000122

**Published:** 2008-08-08

**Authors:** Stefan Magez, Anita Schwegmann, Robert Atkinson, Filip Claes, Michael Drennan, Patrick De Baetselier, Frank Brombacher

**Affiliations:** 1 Division of Immunology, Institute for Infectious Diseases and Molecular Medicine (IIDMM), Health Science Faculty, University of Cape Town, and International Centre for Genetic Engineering and Biotechnology (ICGEB), Cape Town, South Africa; 2 Department of Molecular and Cellular Recognition, VIB, Brussels, Belgium; 3 Laboratory of Cellular and Molecular Immunology, Vrije Universiteit Brussel (VUB), Brussels, Belgium; 4 Laboratory of Serology, Institute for Tropical Medicine “Prins Leopold”, Antwerpen, Belgium; University of Massachusetts Amherst, United States of America

## Abstract

African trypanosomes are extracellular parasitic protozoa, predominantly transmitted by the bite of the haematophagic tsetse fly. The main mechanism considered to mediate parasitemia control in a mammalian host is the continuous interaction between antibodies and the parasite surface, covered by variant-specific surface glycoproteins. Early experimental studies have shown that B-cell responses can be strongly protective but are limited by their VSG-specificity. We have used B-cell (µMT) and IgM-deficient (IgM^−/−^) mice to investigate the role of B-cells and IgM antibodies in parasitemia control and the *in vivo* induction of trypanosomiasis-associated anemia. These infection studies revealed that that the initial setting of peak levels of parasitemia in *Trypanosoma brucei*–infected µMT and IgM^−/−^ mice occurred independent of the presence of B-cells. However, B-cells helped to periodically reduce circulating parasites levels and were required for long term survival, while IgM antibodies played only a limited role in this process. Infection-associated anemia, hypothesized to be mediated by B-cell responses, was induced during infection in µMT mice as well as in IgM^−/−^ mice, and as such occurred independently from the infection-induced host antibody response. Antigenic variation, the main immune evasion mechanism of African trypanosomes, occurred independently from host antibody responses against the parasite's ever-changing antigenic glycoprotein coat. Collectively, these results demonstrated that in murine experimental *T. brucei* trypanosomiasis, B-cells were crucial for periodic peak parasitemia clearance, whereas parasite-induced IgM antibodies played only a limited role in the outcome of the infection.

## Author Summary

African trypanosomiasis is a disease caused by different species of extracellular flagellated protozoan trypanosome parasites. Trypanosomes have developed a mechanism of regular antigenic variation of their variant-specific surface glycoprotein (VSG) coat which allows chronic infection. Replacement of this coat occurs at rapid regular time intervals, allowing the parasite to escape from an effective host antibody responses. So far, primary T-cell independent antibody responses have been described to constitute the main host defense mechanism, relying largely on IgM antibody induction. Using genetically engineered B lymphocyte- or IgM-deficient mouse strains, we show that lack of B-cells or IgM did not prevent infection-associated anemia. More importantly, we show that in the absence of IgM, parasitemia was controlled almost as well as in wild-type mice, with only slightly increased mortality. In addition, we show *in vivo* that antigenic variation is not affected by the lack of IgM.

## Introduction

African trypanosomes are extracellular protozoa that cause chronic infections in humans and livestock and are predominantly transmitted by the bite of the haematophagic tsetse fly [1]. *Trypanosoma brucei gambiense* and *Trypanosoma brucei rhodesiense* are the causative agents of West/Central- and East-African Sleeping Sickness respectively, also called Human African Trypanosomiasis (HAT), and are responsible for an estimated 500,000 infection cases *per annum* [2]. *Trypanosoma congolense*, *Trypanosoma vivax* and *Trypanosoma b. brucei* are considered the main cause for livestock infections. These infections have striking effects on economic growth, with losses exceeding 1 billion US $/year in Africa [3]. Livestock trypanosomiasis moreover affect public health, as infected animals serve as a reservoir for tsetse transmission to humans [2],[4].

The main mechanism generally considered to mediate parasitemia control in a mammalian host, is the continuous interaction between antibodies and the parasite surface, covered by variant-specific surface glycoproteins (VSG) [5]. Trypanosomes undergo antigenic variation by either changing VSG expression sites, known as *in situ* switching of transcriptional control, or by gene replacement resulting in a switch of the terminal telomeric VSG gene itself [6],[7].

Studies in experimental rodent infection models have implicated T-cell-independent anti-VSG IgM responses to be the first line of host defence against proliferating parasites [8]. Experimental approaches using mice depleted of B-cells by polyclonal antibody treatment [9], or infections followed by drug-treatment [10], have shown that B-cell responses can be strongly protective but are limited by their VSG-specificity. This has recently been confirmed in a Cape Buffalo model for natural trypanosomiasis resistance [11]. Recently however, using a chimera bovine model, it was shown that trypanosomiasis sensitivity or resistance was not solely linked to the haematopoietic background of the host, suggesting that other additional host derived factors might also play an important role in the determination of bovine resistance phenotypes [12].

Apart from immune mediated control of infection, the initial setting of parasitemia levels and waves of successive parasitemia peaks are regulated by trypanosomes themselves. This coincides with the differentiation of actively dividing ‘long slender’ parasites into non-dividing ‘short stumpy’ parasites [1], [13]–[15]. In experimental murine trypanosomiasis infection models, pleomorphic parasite populations consist of both ‘long slender’ and ‘short stumpy’ differentiation forms whereas monomorphic populations consist of ‘long slender’ forms only. The latter is highly virulent and kills mice rapidly due to the exponential growth of the ‘long slenders’. The interaction between the different trypanosome forms and the host immune system can therefore be studied by performing experimental infections using pleomorphic and monomorphic trypanosomes that upon infection initially express the same VSG coat (clonal).

One of the most detrimental consequences of trypanosomiasis is anemia, which has been described in experimental mouse models [16]–[18] and livestock [19],[20]. In cattle, monitoring of the dramatic decrease in packed red cell volume (PCV) is the main tool for diagnosis of animal trypanosomiasis, only followed later by parasite detection in the circulation [21]. In human infections, in particular during the hematolymphatic stage of disease, blood and serum anomalies including anemia are also commonly present [22]. However, there is currently a lack of data to explain the occurrence of trypanosomiasis-associated anemia. Some studies have suggested infection-induced anti-VSG antibodies are involved in an erythrolytic process [23], whereas other studies have suggested that trypanosomes release toxic components which directly lyse red blood cells (RBC) [24]. However, we have recently shown that the severity of anemia did not correlate with the actual parasite load [18].

Considering (i) the limited knowledge of the role of individual antibody isotypes in trypanosomiasis control, and (ii) the unclear role of B-cells in the *in vivo* induction of trypanosomiasis-associated anemia, we used B-cell (µMT) and IgM-deficient (IgM^−/−^) mice to address these points. Our results showed that although B-cell- and IgM-deficient mice infected with the clonal *T. brucei* AnTat 1.1E parasites exhibited a reduced life span and impaired parasitaemia clearance, infection-induced IgMs played only a limited role in host survival during non-clonal infections. In addition, the presence or absence of B-cells or infection-induced IgM antibodies did not change the rate of trypanosome surface antigenic variation nor did it affect the occurrence of infection-associated anemia. Therefore, our results show that in mice the overall importance of the infection-induced IgM response is limited.

## Materials and Methods

### Animals

Eight to 12 week old female BALB/c mice, BALB/c µMT [25] mice and IgM-deficient BALB/c (IgM^−/−^) mice [26], as well as C57BL/6 and C57BL/6 µMT mice were obtained from an SPF breeding facility at the University of Cape Town. All mice were housed in filter-top cages and maintained in SPF barrier facilities in individual ventilated cages at the University of Cape Town, or at the Institute for Tropical Medicine Antwerp.

Tsetse flies *(Glossina morsitans morsitans)* were available from the insectaria at the Prins Leopold Institute of Tropical Medicine Antwerp (ITMA), originating from puparia collected in Kariba (Zimbabwe) and Handeni (Tanzania). Flies were fed on rabbits and maintained at 26°C and at a relative humidity of 65%. Animal ethics approval for the tsetse fly feeding on live animals was obtained from the Animal Ethical Committee of the Institute of Tropical Medicine, Antwerp (Belgium).

### Parasites

The *T. brucei brucei* AnTat (Antwerp Trypanosoom antigen type) 1.1E, the *T. brucei brucei* AnTat 1.1 and the non-cloned reference *T. brucei* TSW196 parasite stocks were kindly provided by Dr. N. Van Meirvenne and E. Magnus (Lab. Serology, Institute of Tropical Medicine (ITG), Antwerp, Belgium) and the MITat 1.2 and 1.4 parasite stocks by Dr. M.A.J. Ferguson (Dept. of Biochemistry, The University of Dundee, Scotland). The *T . b. brucei* AnTat 1.1 clonal, monomorphic parasite strain was originally generated as a daughter clone of the AnTat 1.1E clonal, pleomorphic parasite strain. This was done by 25 cycles of syringe passing on day 4 of infection. Due to the short interval between passages, the AnTat 1.1 parasite is highly virulent and expresses only one type of VSG throughout infection, which is homologous to the initial VSG expressed by the AnTat 1.1E clone. Similarly, the *T. brucei brucei* MITat (Molteno Institute Trypanozoon antigen type) 1.2 and 1.4 clonal, monomorphic parasites are highly virulent and express only one VSG that is non-homologous to the initial VSG expressed by the AnTat 1.1E clone. The TSW196 *T. b. brucei* non-clonal, pleomorphic parasite stock was isolated in 1978 in Côte d'Ivoire, from infected pig and was transferred to an OF1 mice in order to make stabilates for storage at −80°C. It is catalogued at the ITG as ITMAS300500A. The AnTaR 1 (Antwerp Trypanozoon antigen repertoire) non-clonal, pleomorphic parasite strain was isolated in 1966 in Uganda from the bloodstream of infected bushbuck. Pleomorphic parasite populations are defined as consisting of both ‘long slender’ and ‘short stumpy’ differentiation forms, whereas monomorphic parasite populations consist of only ‘long slender’ forms. The terms clonal and non-clonal indicates that the parasite population used to infect mice initially express identical or multiple, different VSGs respectively.

### Infection re-challenge, parasitemia & red blood cell counts

Infections were initiated by intraperitoneal (i.p.) injection of 5×10^3^ parasites, unless stated otherwise. Parasites and red blood cells in a 2.5 µl blood sample 1/200 diluted in PBS, were counted every 2 to 4 days, using a light microscope. For super-infection experiments, mice were initially infected with 5000 *T. brucei* AnTat 1.1E parasites by i.p. injection. On day 6 or day 10 post primary infection, mice were super-infected with 5000 homologous highly virulent monomorphic AnTat 1.1 parasites or equally virulent monomorphic MITat 1.4 parasites. These monomorphic parasites were capable of expressing only one type of VSG during infection which were either homologous (AnTat 1.1) or non-homologous (MITat 1.4) to the VSG initially expressed during primary infection. For tsetse fly challenge experiments, freshly emerged tsetse flies were infected by feeding on AnTaR 1 infected mice at the peak of parasitemia. In order to obtain a pleomorphic trypanosome population at high titer, these mice were immune suppressed with cyclophosphamide (20mg/kg). 28 days after the infecting blood meal, flies were screened for a mature salivary gland infection by induced probing on pre-warmed glass slides followed by a microscopic analysis for the presence of metacyclic trypanosomes in the saliva. Infection of tsetse flies with *T. brucei* parasites was performed in compliance with the regulations for biosafety and under approval from the Environmental administration of the Flemish government. To initiate a natural infection, one individual tsetse fly with a mature salivary gland infection was allowed to feed per mouse. To avoid interrupted tsetse feeding, mice were anesthetized prior to the tsetse exposure. Parasite burden of all mice was monitored every day for 1 week following super-infection and survival of all mice was recorded. The experiment was performed in WT BALB/c mice as well is in BALB/c µMT, and IgM^−/−^ mice. 10 mice per group were used.

### Anti-VSG antibody titer determination by ELISA

Infection-induced anti-VSG titers were determined by ELISA as described before [27]. Briefly, anti-VSG mouse antibody isotypes were detected using biotin coupled goat-anti-mouse Ig isotype antibodies (Pharmingen) in combination with streptavidin alkaline-phosphatase (AP) for development. Optical density was determined at 450 nm using a VERSAmax ELISA reader (Molecular Division).

### Quantitative real time RT-PCR

Washed DE52 purified trypanosomes were lysed in TriReagent (Molecular Research Centre) and total RNA extracted according to the manufacturer's instructions. Contaminating genomic DNA was digested using molecular grade RNase-free DNAse I (Promega). Reverse transcriptase and cDNA quantification by real-time PCR on the Lightcycler (Roche Diagnostics) was performed as recently described [28] using the DNA Master Hybridization Probe Kit (Roche Diagnostics) according to the manufacturers instructions. Amplification primers used were: VSG AnTat 1.1E (GenBank accession X01843): (forward 5′-GAA TGC GAC ACG GAA AGC G-3′, reverse 5′-CGT CGT TGG CTG CTT GGA G; 399 base pair product), VSG MITat 1.2 (forward 5′-ATG GAC ACC AGC GGA ACA AAC-3′, reverse 5′-TCC AGG CGT CGA TCC ACG-3′; 259 base pair product), gene tubulin zeta (GenBank accession AF241275) (forward 5′-TCC CGT CCA TTT CAG GTC C-3′, reverse 5′-GTG CAT CAG CAT ACC ATC CAG T-3′; 294 base pair product). Specific hybridization probes for the three genes were respectively VSG AnTat 1.1E flourescein labeled 5′-CTG CT TGC TTG TAG GTG CTG CCG-3′, LC640 5′-CGT TAC AGT TGC CAG TTT AGC TGC GA-3′, VSG MITat 1.2 (GenBank accession X56762) flourescein labeled 5′-TAG CGA ACA GCC AAA CAG CCG TCA C-3′, LC705 5′- GTC CAG GCG CTC GAT GCA TTA CAG-3′, and (Tubulin zeta) flourescein labeled 5′-TGC CTG TAC CAC CAG CTA AAC TGT GT-3′, LC640 5′-TAC CAA AAT TGC CTC AAA CTC CTC CG-3′). A standard curve for AnTat 1.1E, MITat 1.2 and tubulin zeta, was established by 10-fold dilutions of a positive sample and included as a standard and a “calibrator control”. Target mRNA levels in IgM^+/+^, µMT and IgM^−/−^ derived trypanosomes were determined by comparing the sample threshold cycle number against the target gene standard curve using the Second Derivative Maximum function of the Lightcycler software (Roche Diagnostics). The same dilutions of the samples were used for the AnTat 1.1E, MITat 1.2 and tubulin zeta RT-PCR. AnTat 1.1E VSG levels for each sample ware normalized by dividing the calculated AnTat 1.1E value by the calculated tubulin zeta value. The MITat 1.2 RT-PCR served as negative control on AnTat 1.1E derived mRNA. Fluorescent data for each sample was detected at the annealing step at 60°C and hybridization probe specificity cross-checked.

### Graphic and statistical result analysis

All graphic result presentations were prepared using GraphPad Prism software. The same software was used for statistical analysis of data. Comparative analysis of survival data was done using a designated GraphPad Prism statistical module using a Logrank test.

## Results

### B-cells are essential to prolong survival during African trypanosomiasis

**Figure 1:**
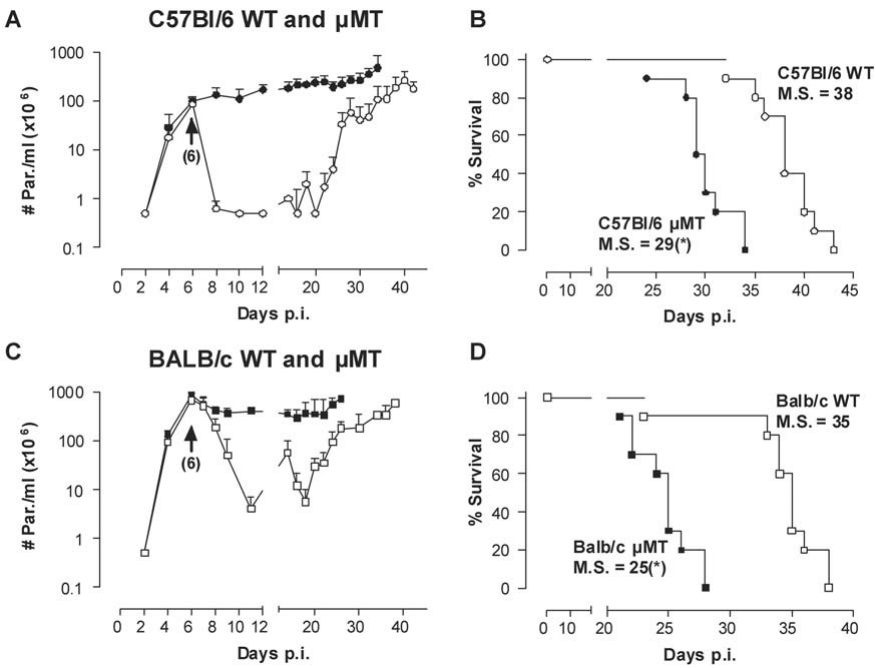
The effect of B-cells in trypanosomiasis control of clonal infections. (A) C57BL/6 WT (○) and µMT (•) mice were infected with a pleomorphic *T. brucei* AnTat 1.1E clone by intra-peritoneal inoculation of 5000 parasites. Parasitemia was followed by microscopy analysis of tail-cut blood samples. Values are presented as mean±SD of 10 individual mice per group. (B) Mortality of all infected mice was recorded. One representative from 3 experiments is shown. (C,D) The same experiment was performed with BALB/c WT (□) and µMT BALB/c (▪) mice. M.S. = median survival time in days. (*: p<0.0001 compared to WT)

To address the role of B lymphocytes during experimental trypanosomiasis, comparative infection studies were performed in B-cell-deficient (µMT) and WT C57BL/6 mice, using the pleomorphic *T. b. brucei* AnTat 1.1E clone. This parasite clone has been used in the past as a well established model for experimental trypanosomiasis in mice, and produces a relatively chronic infection that lasts usually over a month, before killing the host. The pleomorphic annotation refers to the fact that blood parasite populations comprise both proliferating ‘long slender’ parasites and ‘short stumpy’ non-proliferating parasites, which are awaiting transmission to the tsetse fly vector. Figure 1A shows that in C57BL/6 mice, the absence of B-cells had neither an influence on the initial growth rate of the trypanosomes, nor on the height of the parasitemia at 6 days post infection, corresponding to the first peak in WT mice. Subsequently, post-peak parasite removal and prolongation of the mean survival of the infected mice were clearly B-cell dependent (Fig. 1A, B). Important to note is that even in the absence of any anti-trypanosome antibody response, mice controlled a slowly but gradually increasing level of infection for up to 4 weeks. The eventual death of all µMT and WT mice was associated with exponential parasite proliferation growth, resulting in parasitemia levels up to 2×10^9^ parasites/ml. High parasitemia before death was largely independent of the genetic background of the mice, since comparative experimental infections in WT and µMT deficient mice on either a BALB/c or C57BL/6 background yielded similar results (Fig. 1 C,D).

### Trypanosome-induced IgMs have a limited impact on host survival

**Figure 2:**
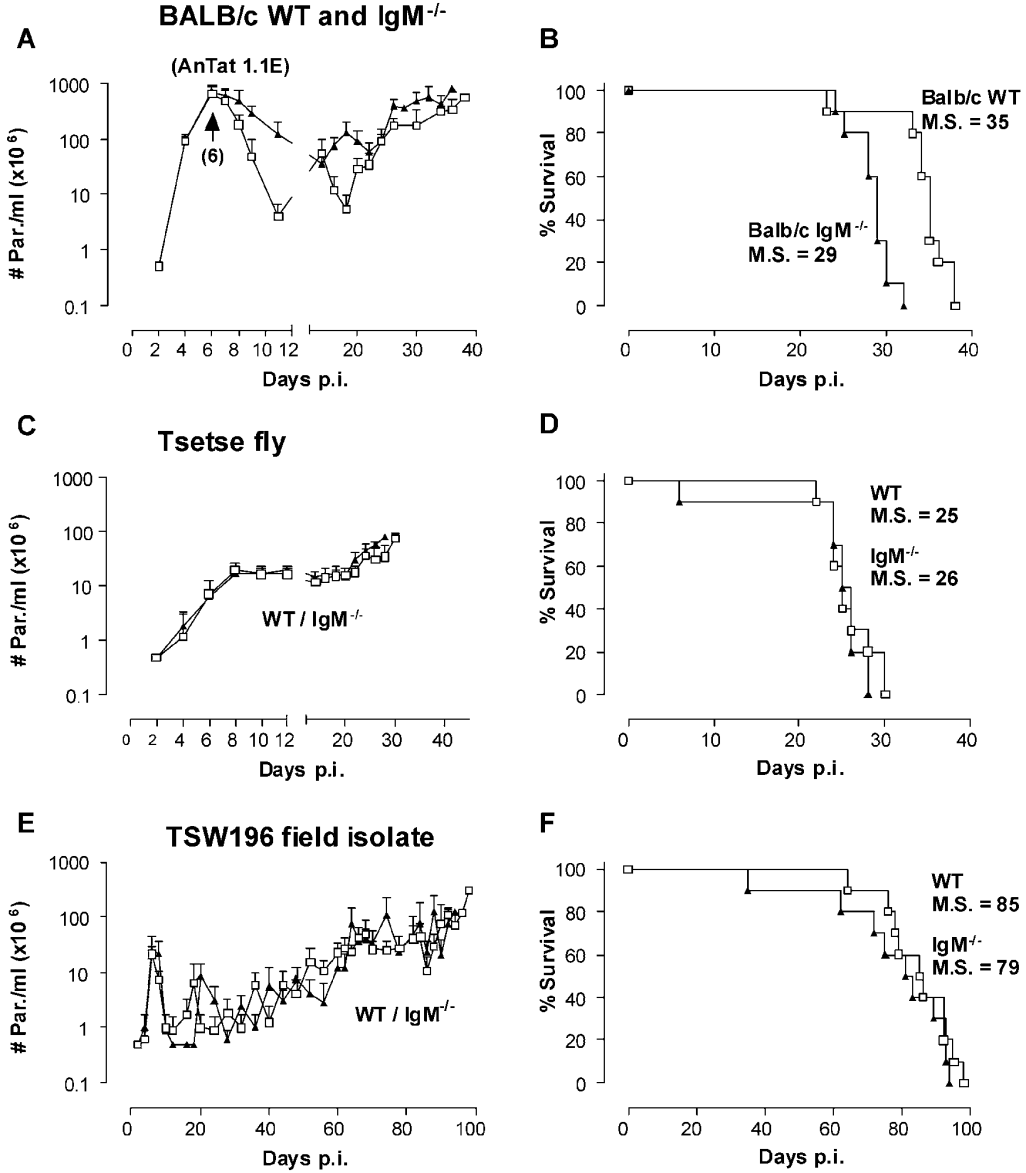
The effect of IgMs in high-, intermediate- or low-virulent infections. BALB/c WT (□) and IgM^−/−^ (▴) mice were infected with (A/B) 5000 parasites of the pleomorphic AnTat 1.1E clone by intra-peritoneal injection, (C/D) non clonal *T. brucei* AnTaR 1 parasites through exposure to tsetse fly or (E/F) with 5000 parasites of the low virulent field isolate TSW196 by intra-peritoneal injection. Parasitemia and mortality of infected mice was assessed as in Fig. 1, using 10 mice per experimental group. M.S. = median survival time.

As parasite VSGs rapidly change, primary immunity provided by T-cell independent IgMs, is believed to confer a crucial impact on trypanosomiasis control [8],[27],[29]. In order to test this *in vivo*, IgM^−/−^ and control WT mice were infected with clonal, pleomorphic *T. b. brucei* AnTat 1.1E parasites as described above. Interestingly, in the absence of IgM, mice were able to reduce peak parasitemia levels, albeit with delayed kinetics and reduced efficacy as compared to WT controls (Fig. 2A). The reduced capacity to clear successive parasitemia waves resulted in a slight, but significant, accelerated mortality (Fig. 2B).

To evaluate whether changes in intrinsic parasite virulence affects the importance of antibodies in trypanosomiasis control, and to mimic natural conditions, tsetse fly infections were used. Surprisingly, the absence or presence of IgM's had no impact on the parasitemia development and survival of mice infected with AnTaR 1 parasites (Fig. 2C and 2D), which are a non-clonal, pleomorphic *T. b. brucei* stock.

In a second attempt to mimic a more natural infection, usually characterized in cattle by a non-clonal, low-level parasitemia, mice were infected with non-clonal, pleomorphic *T. b. brucei* TSW196 parasites derived from a field isolate. When infected with TSW196 parasites, a low virulent infection was obtained in mice. Here again, no significant differences in parasite burden or mortality were observed between WT and IgM^−/−^ mice (Fig. 2E and 2F), suggesting no significant role for IgM responses against the non-cloned *T. b. brucei* TSW196 parasite strain.

### IgM^−/−^ mice produce compensatory soluble IgD antibodies during trypanosome infections

**Figure 3:**
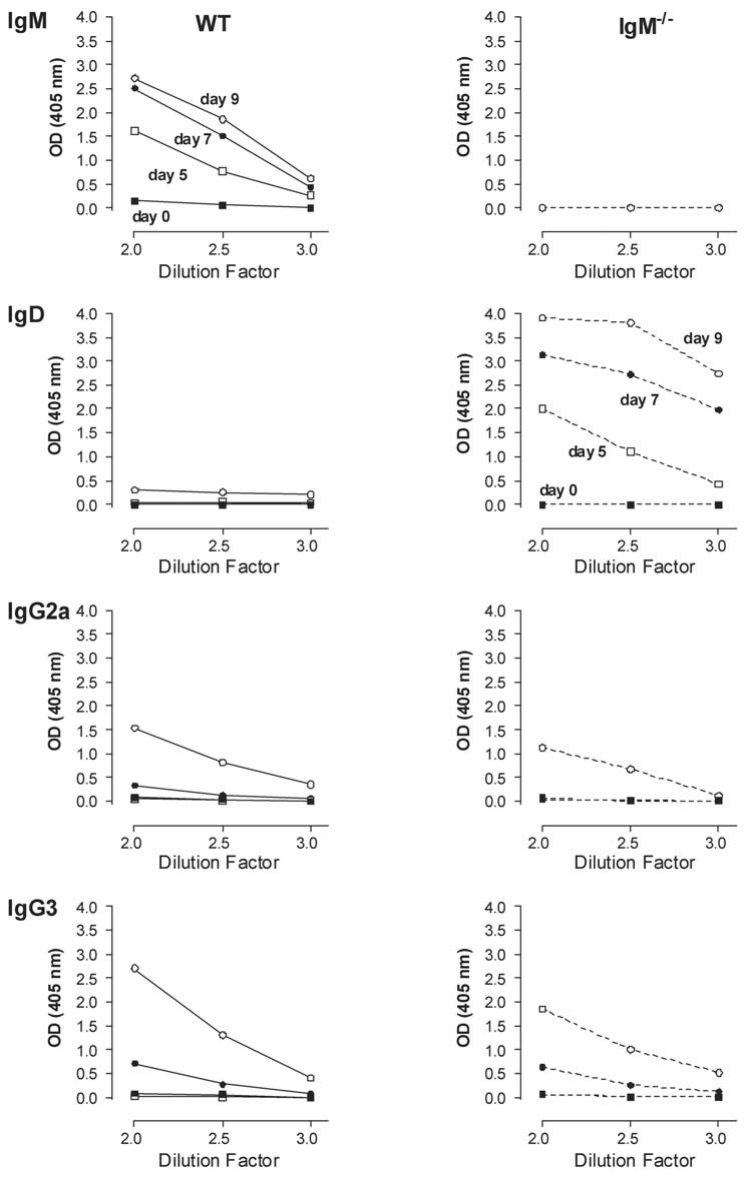
Trypanosome infected IgM^−/−^ mice produce compensatory IgD titers. Sera were collected from *T. brucei* AnTat 1.1E infected WT and IgM^−/−^ BALB/c mice, and were analyzed as serial dilutions (log 10) in a solid phase VSG coated ELISA, using isotype-specific antibodies for detection. Sera were collected throughout the first peak and clearance phase from 5 individual mice. Values are presented as means.

Antibody binding of the parasite surface is considered to be a crucial step in protective host mechanism used to neutralize blood borne parasites. Here, the ability of mice to produce VSG-binding antibody responses was measured in the presence or absence of IgM. In WT mice, infection with clonal, pleomorphic AnTat 1.1E parasites induced rapid VSG-binding IgM antibody serum titers (Fig. 3) with increasing concentrations towards peak parasitemia (day 7). VSG-binding IgG2a and IgG3 antibody titers were detected with a delay of 2 days. Other VSG binding antibody isotypes were hardly detectable (data not shown). A similar kinetic for IgG2a and IgG3 was seen in infected IgM^−/−^ mice. However, in these mice IgM titers were replaced by compensatory VSG-binding soluble IgD titers.

### IgM antibodies protect against a homologous re-challenge infection

Previous studies have suggested that during pleomorphic infections, parasitemia peak clearance involves mainly macrophage-mediated removal of non-dividing ‘short stumpy’ parasites that predominate at this time point [13],[30],[31]. The antibody results presented above may suggest that infection-induced IgMs do contribute to peak parasitemia clearance of clonal parasites. In order to evaluate the functional activity of antibodies in control of actively proliferating ‘long slender’ parasites, an infection model was established using slow killing pleomorphic and fast killing monomorphic ‘long slender’ *T. brucei* clones, expressing either homologous or non-homologous VSGs.

**Figure 4:**
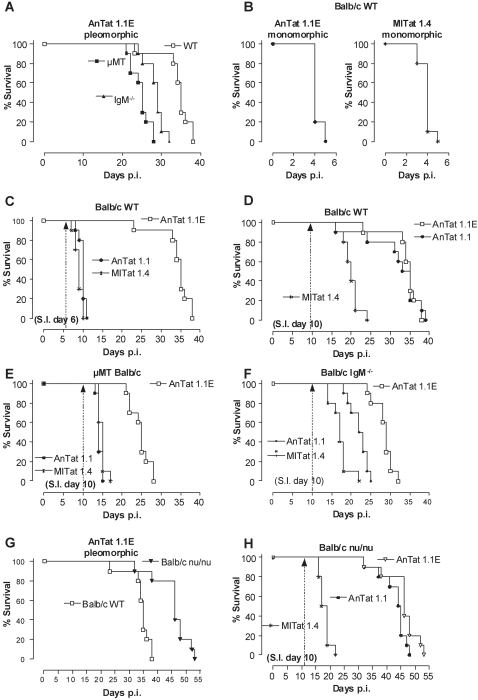
The role of IgM in VSG-specific protection. (A) BALB/c WT (□), IgM^−/−^ (▴) and µMT (▪) mice were infected with 5000 pleomorphic AnTat 1.1E parasites. (B) BALB/c WT mice were infected with 5000 monomorphic AnTat 1.1 (•) or MITat 1.4 (▒) parasites. (C) AnTat 1.1E infected BALB/c WT (□) mice were super-infected (S.I.) on day 6 or (D) on day 10 with 5000 parasites of a homologous, monomorphic AnTat 1.1 (•) or a non-homologous monomorphic, MITat 1.4 (▒) strain. (E) AnTat 1.1E infected BALB/c µMT (□) and (F) BALB/c IgM^−/−^ (□) mice were super-infected with AnTat 1.1 (•) or MITat 1.4 (▒) parasites using the same strategy described above in C–D. (G) BALB/c WT (□) and BALB/c ^nu/nu^ (▾) mice were infected with 5000 AnTat 1.1E parasites and (H) were super-infected AnTat 1.1 (•) or MITat 1.4 (▒) parasites using the same strategy described above in C–D. All primary and super-infections were done by intra-peritoneal inoculation of 5000 parasites. Mortality was recorded using 10 mice per experimental group and the results were compared to mice that only received the primary infection. One out of 3 representative experiments is shown.

First WT, µMT and IgM^−/−^ mice were infected with 5000 AnTat 1.1E parasites, resulting in relatively slow mortality kinetics (Fig. 4A, combining results of Fig. 1D and 2B). In parallel, the same 3 mouse strains were infected with 5000 highly virulent monomorphic AnTat 1.1 parasites (expressing only a VSG that was homologous to the initial VSG of the AnTat 1.1E clone) or with 5000 non-homologous VSG monomorphic MITat 1.4 parasites. Figure 4B shows that both monomorphic parasite clones kill their WT host within 5 days. Identical results were obtained in µMT and IgM^−/−^ mice (data not shown), suggesting that through their accelerated exponential growth, these parasites have reached a level of virulence that is lethal before an *in vivo* efficient B-cell response can be initiated.

Next, a combination infection model using pleomorphic parasites for a primary infection, and monomorphic parasites for a re-challenge infection was established. WT mice were infected with pleomorphic AnTat 1.1E parasites (Fig. 4C) and on day 6 after primary infection, when the parasite load was greater than 10^8^ parasites/ml (data not shown), they were re-challenged with 5000 monomorphic AnTat 1.1 or MITat 1.4 parasites. These monomorphic parasites expressed VSGs that were either homologous (AnTat 1.1) or non-homologous (MITat 1.4) to the initial VSG expressed during primary infection. This resulted in significant earlier mortality of re-challenged mice, as compared to primary infected mice, irrespective of the homology of the VSG being expressed by the parasite during re-challenge (Fig. 4C) (*p*<0.0001). This earlier mortality was triggered by uncontrolled growth of ‘long slender’ forms of the monomorphic clones (Fig. 4C), as mice died with high parasitemia exceeding 10^9^ parasites/ml blood after temporary remission of their primary pleomorphic infection (parasitemia data not shown). However, when re-challenged at day 10 with monomorphic AnTat 1.1 parasites, expressing homologous VSGs, rapid host killing was not observed demonstrating VSG-specific resistance (Fig. 4D). Indeed re-challenge at day 10 with non-homologous VSG expressing parasites (MITat 1.4) resulted in significantly accelerated mortality (M.S. = 18 days) as compared to control WT and AnTat 1.1 infected mice (M.S. = 34 days for both groups) (Fig. 4D). However, the observed host killing occurred with slower kinetics than observed in Fig. 4B. VSG-specific resistance at day 10 was absent in µMT mice (Fig. 4E) which displayed a mean survival of 15 days. This was not unexpected since both monomorphic parasites killed WT mice within 4–5 days (Fig. 4B). VSG-specific resistance in IgM^−/−^ mice re-challenged at the same time point with the same parasite was significantly impaired as compared to WT mice (*p*<0.0001) (Fig. 4D). However, IgM^−/−^ mice infected with AnTat 1.1 survived 1 week longer (Fig. 4F) than the 15 days observed in µMT mice (Fig. 4E). This earlier mortality of IgM^−/−^ mice as compared to WT mice, showed that IgM's play only a minor role in protection against homologous re-challenge infection. In contrast T-cell deficient mice, which would be expected to produce T-cell independent anti-VSG IgM antibodies, were fully protected against homologous challenge (Fig. 4G and 4H).

### Antigenic variation occurs in the absence of antibody responses

**Figure 5:**
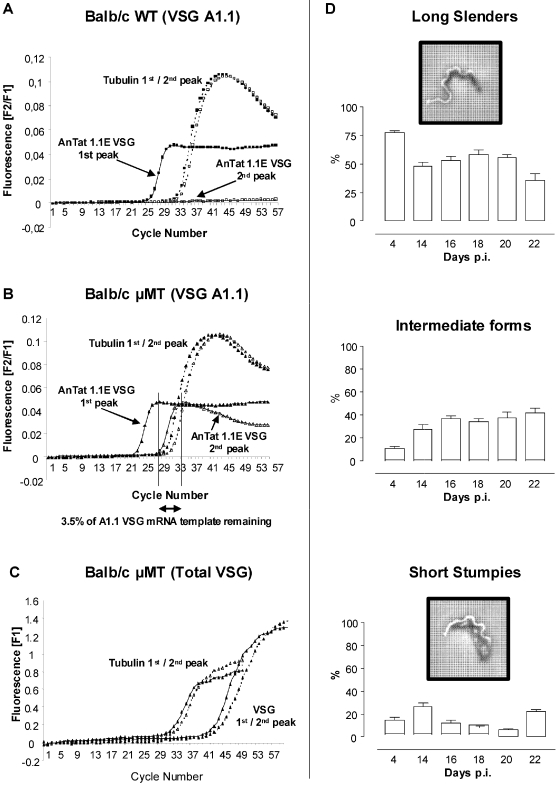
Quantitative analysis of VSG switching. Switching of VSG expression was followed by real time RT-PCR, by amplification of peak stage parasite RNA with primers specific for VSG AnTat 1.1E and the housekeeping genes tubulin-zeta (*Tubulin*). (A) RNA was isolated from WT derived and (B) µMT derived parasites on the first and second peak, respectively occurring on day 6 and 14. Expression of VSG AnTat 1.1E specific RNA on the second peak was calculated and compared to the first peak, after normalization of the first and second peak VSG AnTat 1.1E specific RNA to tubulin-zeta gene expression.. One representative of two experiments are shown. (C) Similarly, total VSG mRNA was amplified from first and second peak µMT derived parasites, using VSG-Uni primers, and results were normalized using *Tubulin* gene expression. (D) To assess parasite proliferation in µMT mice, the occurrence of dividing ‘long slender’ parasites versus intermediate and non-dividing ‘short stumpy’ parasites was analyzed by light microscopy on day 4 and compared to the chronic infection stage (up to day 22).

Antigenic variation is a characteristic feature of trypanosomes allowing escape from protective host immune responses. It is suggested to result in characteristic waves of parasitemia through interplay with anti-VSG-specific antibodies [32]). Interestingly, the presence of VSG switching during *in vitro* cultivation of trypanosomes under selective drug pressure, demonstrated that antigenic variation can occur in the absence of antibody mediated events [33] . However, this is not conclusive for *in vivo* conditions in mammals. Due to B-cell mediated selection pressure, one might expect differences in the overall VSG-specific RNA species. Quantification of VSG AnTat 1.1E specific RNA by Real Time RT-PCR from AnTat 1.1E infected B-cell deficient (µMT) or wild-type mice showed similar VSG AnTat 1.1E specific RNA concentrations during the first parasitemia peak (Fig. 5A, B). Using irrelevant VSG MITat 1.2 specific primers, no amplification signal was obtained under identical real time RT-PCR conditions, demonstrating the VSG specificity of the assay (data not shown). VSG AnTat 1.1E specific RNA was undetectable in parasites isolated from the second peak of parasitemia of WT mice, due to efficient elimination of the VSG AnTat 1.1E parasites. Interestingly in µMT mice, VSG AnTat 1.1E specific RNA from second peak parasites was strikingly reduced despite exponential parasite growth (Fig. 5B), which suggests that the main evasion mechanism is an intrinsic genetic program. The finding of 3.5 % remaining VSG AnTat 1.1E specific RNA in µMT-derived parasites compared to the sensitivity level of >1%, may reflect a slight reduction in efficiency of clonal parasite elimination in the absence of antibody-mediated selection pressure. The possibility that a reduction of VSG AnTat 1.1E specific RNA was due to a proliferation/growth arrest of parasites could be ruled out as the total amount of VSG-specific RNA species using quantification by Uni-primers (able to amplify all VSG RNA species) was similar during the first and second peak, shown in Fig. 5C. This was confirmed by microscopy analysis of infected blood of µMT mice (day 14–22), that contained around 50% dividing ‘long slender’ parasites and 20% non-dividing ‘short stumpy’ parasites (Fig. 5D).

### B-cells are not involved in the induction of trypanosomiasis-associated anemia

**Figure 6:**
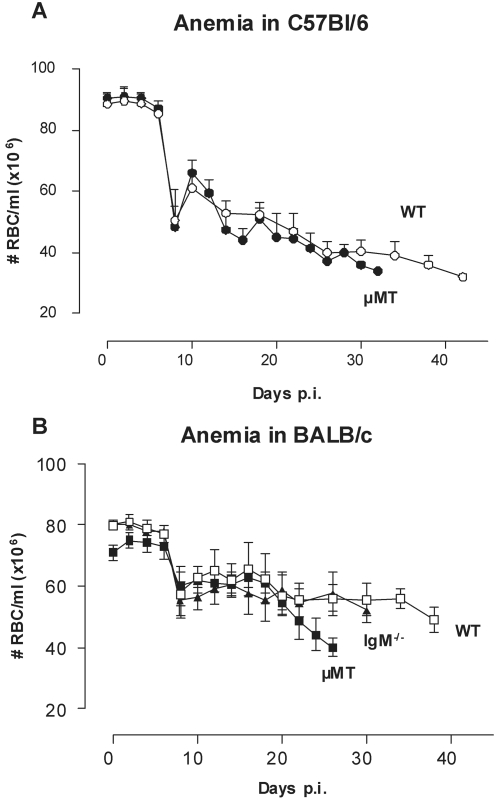
Trypanosomiasis-associated anemia is present in µMT mice. (A) C57BL/6 WT (○) and µMT (•) mice were infected with a pleomorphic *T. brucei* AnTat 1.1E clone by intra-peritoneal inoculation of 5000 parasites. (B) BALB/c WT (□), µMT (▪) and IgM^−/−^ (▴) mice were infected by intra-peritoneal inoculation of 5000 with the pleomorphic AnTat 1.1E clone. RBC counts were followed by microscopy analysis of tail-cut blood samples. Values are presented as mean±SD of 10 individual mice per group.

Infection-induced anemia is an important morbidity factor in African trypanosomiasis and has been linked to B-cell responses, due to lysis of red blood cells after opsonization with VSG-specific antibodies [23]. However, trypanosomiasis-induced anemia was as severe in µMT mice as in WT mice (Fig. 6). This was independent of the mouse genetic background, as similar patterns of anemia development were recorded in C57BL/6 (Fig. 6A) and BALB/c mice (Fig. 6B). Furthermore, trypanosomiasis associated anemia also occurred in the absence of a functional IgM response (Fig. 6B).

## Discussion

African Trypanosomiasis is a textbook example of an extracellular parasitic infection where antigenic variation of the variant-specific surface glycoprotein (VSG) and subsequent antibody responses of the host result in a prolonged period of parasitemia control [1],[3],[4],[32]. For the parasite, this lengthened time is required to increase the chance of successful parasite transmission through the tsetse vector. While resistance to trypanosomiasis in some mammals such as the Cape Buffalo is linked to their capacity to mount an efficient anti-parasite antibody response, trypano-tolerance in cattle can also develop independently of their genetic haematopoietic background [34]. These observations suggest that besides antibodies, additional host factors can contribute to parasite control. During the chronic phase of trypanosomiasis, the continuous interplay between the parasite and its host's immune system results in infection-associated pathology, including anemia [14]–[20], [31]–[35]. Despite the importance of anemia, the exact mechanisms underlying its induction remained unsolved [35]. Some studies, showing that *in vitro* VSG-sensitized RBC can be lysed by VSG-specific antibodies, have suggested that anemia may be linked to B-cell responses and that antibody mediated lysis may be a contributing factor *in vivo* [23]. Other studies have suggested that trypanosomes themselves release components which directly lyse RBC [24]. Based on *in vitro* observations, it was proposed that destruction of RBC's could occur as a result of an active GPI-VSG transfer between trypanosome and erythrocyte membranes, followed by the anti-VSG mediated complement-dependent lysis [23]. However no *in vivo* data was obtained to support this hypothesis. Moreover, we have previously not been able to find any significant correlation between the severity of anemia and parasite load [18]. Using gene deficient mouse models, we have now dissected the role of B-cells and IgM-responses in parasite elimination, host survival, antigenic variation and in *Trypanosoma*-induced anemia.

We have clearly shown that infected µMT mice still developed anemia, suggesting that B-cells are not involved in the induction of trypanosomiasis-associated anemia. This B-cell independent trypanosomiasis-induced anemia may be mediated by TNF [18],[36],[37], although the exact mode of action remains to be elucidated. TNF could have a crucial impact on anemia through its capacity to modulate the activation, growth and the phagocytic potential of macrophages involved in antibody independent erythrophagocytosis [38]–[40], a process which is dependent on IFNγ [41].

Infections with clonal, pleomorphic *T. brucei* parasites (AnTat 1.1E), demonstrated an essential role for B-cells in host protection and corroborated previous B-cell depletion studies [9],[10]. However, IgM was found to play only a limited role in primary infection with clonal, pleomorphic parasites, perhaps due to the presence of VSG-specific IgG2a and IgG3 and compensatory IgD antibodies or other Ig isotypes. Interestingly, our results in BALB/c µMT and C57BL/6 µMT mice showed that the genetic background of the host has an impact on parasite growth and differentiation, independent of the B-cell compartment. Indeed, in C57BL/6 µMT mice the first parasitemia peak was consistently lower than the BALB/c µMT, albeit in both strains the peak is reached at the same time point. Also, since T-cell deficient mice were found to be fully protected against re-challenge with a homologous parasite, the antibody mediated VSG-specific protection observed on day 10 was T-cell independent. However, the significance of the extended survival of the T-cell deficient mice is beyond the scope of this paper.

Re-challenge studies with the homologous monomorphic AnTat 1.1 strain demonstrated that while VSG-specific IgM's were induced early during infection, they were not able to efficiently protect against monomorphic parasites during the first peak of infection. This was evidenced by the impaired protection in wild-type mice when re-infected with the homologous VSG-expressing AnTat 1.1 strain early during infection at day 6 (see Fig. 4C). In contrast, VSG-specific IgM responses were able to fully protect WT mice against re-infection at day 10 after primary infection. However, efficient elimination of actively proliferating monomorphic parasites occurred only when the parasites used for re-challenge expressed a VSG that was homologous to those expressed during primary infection. These observations support the notion that antibodies can only exert their full anti-trypanosome activity in an immunological environment that develops later than day 6. At this early time point, activated macrophages play a crucial role in parasite destruction via the secretion of inflammatory molecules such as nitric oxide (NO) and TNF, and through phagocytosis of damaged and opsonized parasites**.** Although IgM^−/−^ mice survived a few days longer when re-infected on day 10 with the homologous VSG-expressing AnTat 1.1 parasite, as compared to re-infection using non-homologous VSG-expressing MITat 1.4 parasites (Fig. 4F), this prolonged survival in IgM^−/−^ mice (MS = 12 days) was much less pronounced than the prolonged survival obtained in WT mice, where re-challenge on day 10 did not result in accelerated mortality at all (Fig. 4D). This demonstrated that VSG-specific IgD and/or IgG responses were not able to efficiently compensate for IgM immunity, irrespectively of the homology of the VSG being expressed by the parasite during re-challenge. Because MITat 1.4 re-challenged IgM^−/−^ mice (Fig. 4F) survived longer than MITat 1.4 primary infected IgM^−/−^ mice (Fig. 4B), we concluded that a primary host immune responses from day 10, had a negative effect on parasite growth rate, which seems to be VSG-independent. A similar observation has been reported in natural infections where relative resistance or susceptibility to trypanosomiasis in cattle could not always be correlated to the host's capacity of mounting an efficient anti-VSG response. Instead trypano-tolerance was found to be associated with elevated Type I cytokine production (IFNγ & TNF), increased macrophage activation and NO production [34],[42]. Similarly, during initial exponential parasite growth phase, SCID mice displayed a lymphocyte-independent parasite growth regulating mechanism which gradually triggered the differentiation of proliferating ‘long slender’ parasites into non-dividing ‘short stumpy’ forms causing the infection to reach a plateau [13],[43]. This mechanism of B-cell independent parasite control may therefore involve an intrinsic regulation of parasite growth/differentiation by a parasite released short-stumpy inducing factor [44].

In contrast to the results from infection using clonal, pleomorphic parasites, infections with a non-clonal, pleomorphic field isolate (TSW196) showed that IgM^−/−^ mice were able to remit parasitic waves as efficiently as wild-type control mice. Infection-induced IgM's may therefore be compensated for, perhaps by IgG's or other Ig isotypes, during host protective responses. Similarly, no prominent role for IgM was observed in non-clonal tsetse fly initiated infections. However, in this case it should be noted that the AnTaR 1 *T. brucei* parasite used for tsetse fly transmission caused a highly virulent infection in all experimental groups. Moreover, we can not exclude the possibility that WT mice do not make sufficient IgM in response to tsetse fly initiated infections and that IgM's are important for wave remission. These results may also suggest that either tsetse fly saliva components, or other factors involving intrinsic characteristics of tsetse fly transmitted parasites, actively prevent the induction of efficient B-cell responses in mice as a defence mechanism against the host immune system.

Although, previous *in vitro* experiments indicated that trypanosomes in culture may undergo VSG switching in the absence of antibody-mediated pressure [33], this has never been demonstrated *in vivo*. We have shown that antigenic variation occurring *in vivo* is independent of antibody induction or selective pressure. Although clonally infected µMT mice had overall impaired parasite clearance and shortened survival, the virtual absence of VSG AnTat1.1E specific RNA in µMT mice 10 days after first peak of infection, demonstrated that parasites switched their VSG independently of antibody selective pressure. Moreover, the remaining 3.5% VSG AnTat1.1E specific RNA in µMT mice and 3% VSG AnTat1.1E specific RNA in IgM-deficient mice, indicated that antibodies are mostly needed for complete elimination of the remaining non-switched parasites. Interesting to note is the apparently unaltered VSG switching rate between parasites growing in WT and µMT mice. This observation suggests that antibody independent host or parasite factors drive the actual switching process. While the production of a VSG-specific stumpy-inducing factor by the parasite itself appears unlikely, it is feasible that inflammatory mediators such as NO, oxygen radicals or inflammatory cytokines such as TNF and IFNg, produced by activated macrophages or T-cells could impact on the activation of antigenic variation. Future repetitions of the experiments presented here in BALB/c ^nu/nu^ and/or RAG^−/−^ mice could potentially shed more light on the mechanisms at work here.

In summary, the genetic approach of our study using applicable gene deficient mice has allowed us to discriminate the *in vivo* roles of B-cells as well as IgM-responses in parasite growth control, host survival and pathology associated with trypanosomiasis. Our report shows that B-cells are not involved in the induction of trypanosomiasis-associated anemia. Moreover, although infections with the clonal, pleomorphic *T. brucei* AnTat 1.1E parasites in µMT and IgM^−/−^ mice indicated a limited role for infection-induced anti-VSG antibodies in parasitemia control and host survival. Moreover, non-clonal, low-virulent infections originating from field populations, as well as tsetse fly exposure experiments, indicated that IgM responses have no decisive role on either disease progression or host survival. Finally, this study demonstrated that *in vivo* parasite VSG switching operates as an intrinsically programmed genetic process that is independent of B-cell or antibody pressure, with the function of antibodies mainly limited to the elimination of the remaining non-switched parasites.
